# Can machine learning predict PTSD symptoms from trauma narratives of children and adolescents?

**DOI:** 10.1080/20008066.2025.2589709

**Published:** 2025-12-02

**Authors:** Alessandra Giuliani, Tamsin Sharp, Yeukai Chideya, Richard Meiser-Stedman, Mark Tomlinson, Sarah L. Halligan

**Affiliations:** a Department of Psychology, University of Bath, Bath, UK; b Department of Global Health, Institute for Life Course Health Research, Stellenbosch University, Stellenbosch, South Africa; c Department of Clinical Psychology & Psychological Therapies, Norwich Medical School, University of East Anglia, Norwich, UK; d School of Nursing and Midwifery, Queen's University Belfast, Belfast, UK

**Keywords:** Machine learning, PTSD, trauma narratives, children and adolescents, LLMs, predictive models, Aprendizaje automático, TEPT, narrativas del trauma, niños y adolescentes, modelos de lenguaje grandes (LLMs), modelos predictivos

## Abstract

**Background:** Machine learning approaches are being increasingly tested as a potential means of identifying mental health conditions. Narrative features of trauma memories are proposed to play a significant role in the development of post-traumatic stress disorder (PTSD), meaning that trauma narratives provide an excellent context in which to test machine learning capabilities. The potential for children’s trauma narratives to predict post-traumatic stress remains particularly poorly studied. Here, we tested whether the application of machine learning to trauma narrative characteristics can predict PTSD symptoms in young individuals exposed to trauma.

**Study methodology:** Two pre-trained large language models and two benchmark models were fine-tuned and trained to predict PTSD symptom severity from children’s autobiographical narratives of a traumatic event. Data comprised narratives collected one month post-trauma from 400 individuals aged 7–17 years old who experienced a psychological trauma that led to attendance at emergency departments in the United Kingdom (*N* = 178) and South Africa (*N* = 222), as well as self-reported PTSD symptoms and trauma memory features.

**Findings**: Both pre-trained and benchmark models demonstrated poor predictive performance across trauma narratives in the United Kingdom, South Africa, and the combined datasets (e.g. RoBERTa *R*²  =  –.05; LASSO *R*² ≈ 0). However, adding self-reported trauma memory features, disorganisation, and sensory vividness improved the benchmark models’ performances, especially in the UK dataset (e.g. LASSO *R*² = .57; XGBoost *R*² = .45).

**Conclusions:** These findings indicate that while trauma narratives alone offer limited predictive value, incorporating self-reported trauma memory characteristics substantially enhances model performance, highlighting the importance of focusing on subjective reports to develop scalable automated tools for PTSD risk prediction in youth.

## HIGHLIGHTS

- **Trauma narratives alone offer limited predictive value for PTSD in youth.** Advanced machine learning models, including large language models, failed to predict post-traumatic stress symptoms based on children’s and adolescents’ trauma accounts.
- **The study used trauma narratives from children in both the UK and South Africa.** Despite cultural and linguistic differences, predictive performance was similarly limited across both samples, suggesting a broader developmental challenge in detecting PTSD through narrative language.
- **Subjective memory features substantially enhance predictive performance.** Incorporating self-reported ratings of memory disorganisation and sensory vividness led to significant improvements in model accuracy.

## Abstract (es)

**Antecedentes:** Los enfoques de aprendizaje automático se están probando cada vez más como un posible medio para identificar condiciones de salud mental. Se propone que las características narrativas de los recuerdos traumáticos desempeñan un papel significativo en el desarrollo del TEPT, por lo que las narrativas del trauma ofrecen un contexto excelente para poner a prueba las capacidades del aprendizaje automático. El potencial de las narrativas del trauma en niños para predecir el estrés postraumático sigue siendo un área poco estudiada. En este estudio, probamos si la aplicación del aprendizaje automático a las características de las narrativas del trauma puede predecir los síntomas de TEPT en jóvenes expuestos a trauma.

**Metodología:** Se ajustaron y entrenaron dos modelos de lenguaje grandes preentrenados y dos modelos de referencia para predecir la gravedad de los síntomas de TEPT a partir de narrativas autobiográficas de un evento traumático contado por niños. Los datos incluyeron narrativas recopiladas un mes después del trauma de 400 individuos de entre 7 y 17 años que habían experimentado un trauma psicológico que los llevó a acudir a servicios de urgencias en el Reino Unido (*N *= 178) y Sudáfrica (*N *= 222), además de auto-reportes de síntomas de TEPT y características de la memoria del trauma.

**Hallazgos:** Tanto los modelos de lenguaje preentrenados como los modelos de referencia mostraron un bajo rendimiento predictivo en las narrativas del trauma del Reino Unido, Sudáfrica y en los conjuntos de datos combinados (por ejemplo, RoBERTa *R*² = –,05; LASSO *R*² ≈ 0). Sin embargo, al añadir características auto-reportadas de la memoria del trauma – como la desorganización y la vividez sensorial – se mejoró el rendimiento de los modelos de referencia, especialmente en el conjunto de datos del Reino Unido (por ejemplo, LASSO *R*² = ,57; XGBoost *R*² = ,45).

**Conclusión:** Estos hallazgos indican que, aunque las narrativas del trauma por sí solas ofrecen un valor predictivo limitado, la incorporación de características auto-reportadas de la memoria del trauma mejora sustancialmente el rendimiento de los modelos. Esto resalta la importancia de centrarse en los reportes subjetivos para desarrollar herramientas automatizadas y escalables que permitan predecir el riesgo de TEPT en jóvenes.

## 1. Introduction

Recent advances in machine learning (ML) are reshaping psychological science, enabling novel approaches to mental health screening and risk prediction at scale. ML encompasses computational techniques that enable models to learn from data and generate predictions (LeCun et al., 2015). In mental healthcare, ML holds promise for augmenting clinical workflows, reducing diagnostic burden, and expanding access to care. Prior research has explored its utility in detecting mental health disorders (Zhang et al., 2022) and supporting clinical decision-making (Fu et al., 2023).

Among the most widely used ML paradigms in psychiatric research is supervised learning (Zhou et al., 2020). In supervised learning, models are trained on labelled datasets, where input data are paired with known outcomes. Through iterative learning, the model identifies patterns and generalises them to new, unseen data. This approach has proven effective in applications such as detecting depression from social media text (Coppersmith et al., 2014a), predicting anxiety from clinical interviews (Calvo et al., 2017), and assessing suicide risk using linguistic cues (Zhang et al., 2022). These applications demonstrate the potential of ML-driven approaches to enhance mental health screening and intervention strategies. Fine-tuning large pre-trained language models (LLMs) has emerged as a powerful supervised learning approach for text-based tasks, including sentiment analysis, text classification, and clinical diagnosis. By leveraging vast amounts of pretraining data, LLMs capture rich semantic and contextual representations of language that surpass traditional natural language processing (NLP) techniques. Yet their potential remains underutilised in clinical psychiatry, especially in detecting complex disorders like post-traumatic stress disorder (PTSD) from narrative data.

PTSD is a serious and often underdiagnosed condition in trauma-exposed populations, including children and adolescents (Alisic et al., 2012). Narrative descriptions of trauma experiences, particularly how individuals recall and structure their memories, have been proposed as indirect markers of PTSD symptoms (Crespo & Fernández-Lansac, 2016; Meiser-Stedman et al., 2019a). Specifically, narrative components identified as potential indicators of PTSD include disorganisation (reflecting fragmented and incoherent memory recall), reduced coherence, heightened sensory-perceptual descriptions, alterations in emotional tone, and differences in lexical richness (Crespo & Fernández-Lansac, 2016; Meiser-Stedman et al., 2019b; Rubin et al., 2008, 2016). Among these, disorganisation and sensory detail have shown the most consistent predictive associations with PTSD severity.

These empirical findings align with theoretical accounts of PTSD, emphasising that trauma narratives capture central cognitive and emotional processes implicated in the disorder (Brewin, 2001; Ehlers & Clark, 2000). According to these theoretical perspectives, fragmented autobiographical memory, difficulties with emotional regulation, and disruptions in sensory integration should manifest in how traumatic experiences are recounted (Brewin, 2014).

We focus on children and adolescents because PTSD is both highly prevalent and underdiagnosed in this age group (Alisic et al., 2014; Miele & O’Brien, 2010), and because developmental factors shape how memories are encoded and recalled (Fivush, 2011; Nelson & Fivush, 2004), raising the unresolved question of whether these theoretical perspectives extend to youth trauma narratives. While some studies have identified PTSD-specific linguistic patterns in child populations, such as increased sensory-perceptual descriptions and a greater presence of disorganisation (O’Kearney & Perrott, 2006; Rubin et al., 2008, 2016), others have failed to replicate the same findings (Crespo & Fernández-Lansac, 2016; McGuire et al., 2021). This variability underscores the need for systematic, computational approaches to clarify whether narrative features are predictive of PTSD in youth populations.

Moreover, most prior work on trauma narratives has relied on manual or qualitative analysis (Bryant et al., 2007; Foa et al., 1995; McGuire et al., 2021). More recently, ML approaches have demonstrated impressive accuracy in PTSD classification, particularly when integrating high-dimensional data sources such as biomarkers and structured assessments (Schultebraucks et al., 2022a, 2022b), achieving up to 0.89 classification accuracy in meta-analytic estimates (Wang et al., 2024). However, obtaining multimodal biomarkers such as facial, vocal, and physiological features often requires specialised equipment, controlled conditions, and raises privacy concerns. By contrast, trauma narratives can be collected more easily, making them especially practical for scalable deployment. Importantly, trauma narratives also provide a direct means of testing theoretical accounts of PTSD, as they capture disruptions in memory coherence, emotional regulation, and sensory integration that are central to the disorder’s underlying mechanisms. To date, however, no study has systematically evaluated whether advanced ML models, such as LLMs, can classify PTSD severity based solely on trauma narratives. Existing computational psychiatry studies have focused on social media posts, structured clinical interviews, and electronic health records (Zafari et al., 2021), leaving open the question of whether free-text trauma memories can serve as reliable diagnostic input. Moreover, most prior ML studies in this domain have relied on adult, Western datasets, raising concerns about developmental and cross-cultural generalizability, particularly given that youth in non-Western settings may differ in how they narrate trauma, express emotion, and report symptoms. This underscores the need for more inclusive and representative approaches.

To address these critical gaps in the literature, we systematically investigated whether trauma memory narratives can predict PTSD symptom severity in children and adolescents using ML techniques, applied as follows. First, we employed LLMs fine-tuned to learn deep contextual representations directly from raw text. Next, we used benchmark models trained on features derived from the trauma narratives based on prior studies linking narrative qualities, such as coherence, emotional tone, lexical richness, and perceptual detail, with PTSD symptoms. This dual approach enabled us to directly examine the trade-off between interpretability, offered by benchmark models grounded in theory-driven features, and LLMs’ greater predictive power and linguistic sensitivity. Finally, to further validate the benchmark models, we incorporated self-reported trauma memory characteristics, specifically disorganisation and sensory information scores, which have shown more consistent associations with PTSD symptoms across youth samples than linguistic narrative features. Through this approach, we tested the added value of including narrative features above self-report in PTSD prediction. We additionally assessed the generalisability of all models across different cultural contexts, a critical consideration for real-world use, by utilising datasets from the United Kingdom and South Africa, both independently and combined. We thus aimed to inform future efforts to develop scalable, low-burden tools for identifying PTSD risk in diverse child and adolescent populations.

## 2. Methods

### 2.1. Data

This study uses secondary data from three methodologically similar cohorts whose primary aim was to investigate mental health outcomes in children and adolescents following a traumatic event, as defined by the DSM-5 (American Psychiatric Association, 2013), that required emergency department care. DSM-5 Criterion A traumatic exposure was established from hospital and emergency department records and a brief pre-assessment interview in which caregivers (and the child, where appropriate) reported the event type and details. The studies encompassed a broad range of trauma types, both interpersonal and accidental. Across all studies, within 2–6 weeks post-trauma, participants were interviewed and asked to remember and narrate their traumatic experience, in addition to completing clinical assessments measuring the severity of their PTSD symptoms and their self-reported memory characteristics.

Participants exceeding 20% missingness on any PTSD or trauma memory scale were excluded. When missingness was ≤20% for a given scale, total scores were computed using within-measure mean substitution, replacing each missing item with the participant’s average of their completed items for that scale. This was a pragmatic approach selected to preserve each participant’s symptom profile without drawing on data from others, and it is consistent with simulation work indicating that simple item-level methods perform comparably to more complex approaches when item-level missingness is low (Graham, 2009).

*UK Dataset.* Two UK studies provided 178 participants. The ASPECTS study recruited children and adolescents aged 8–17 (Meiser-Stedman et al., 2019b) and conducted assessments 2–4 weeks post-trauma; 57 participants had both trauma narratives and PTSD data. The PROTECT study (Hiller et al., 2018) recruited 132 children aged 6–13 and assessed participants 2–6 weeks after emergency department attendance; 121 participants had complete data for the current study.

*South Africa Dataset.* The Sinethemba Study, conducted in South Africa, recruited 250 children aged 8–16 identified in hospitals within the Khayelitsha township in Cape Town (Sharp et al., 2024). Participants were interviewed within four weeks post-trauma. All measures were administered in Xhosa, the primary language of the local community. In total, 222 participants had complete narrative and PTSD data.

#### 2.1.1. Narratives

Across all studies, participants were asked to recount the traumatic event they experienced, following near-identical instructions. Narratives were recorded and manually transcribed. In the Sinethemba study, transcripts were translated from Xhosa into English by local bilingual researchers, ensuring accuracy and cultural nuance. Data collectors subsequently reviewed translated transcripts to ensure consistency with the original account and across transcriptions. Transcription approaches were reviewed across all studies, and discrepancies, such as variations in anonymisation approaches, were standardised. Only participants’ free speech was retained, with all interviewer input and transcriber comments removed.

#### 2.1.2. PTSD measures

PTSD symptoms were assessed between 2 and 6 weeks post-trauma using the following established clinical measures.

*UCLA-RI* (*University of California at Los Angeles Reaction Index;* Pynoos et al., 1998). The UCLA-RI was employed by PROTECT study. It is a 20-item self-report questionnaire designed for children and adolescents. It assesses the presence of traumatic experiences and the frequency of PTSD symptoms using a 5-point Likert scale ranging from 0 (‘none of the time’) to 4 (‘most of the time’), resulting in a total symptom score spanning from 0 to 80.

*CPSS-5-SR (Children’s PTSD Symptom Scale Self-Report*; Foa et al., 2018). The CPSS-5-SR, administered in the Sinethemba study, assesses PTSD symptom frequency in children aged 8–18 years. It consists of 20 symptoms rated on a scale from 0 (‘not at all or only once’) to 3 (‘five or more times per week / almost always’), summed to compute a total symptom severity score ranging from 0 to 60. The final seven items measure the impact of PTSD symptoms on daily functioning and do not contribute to the overall PTSD severity score. In the ASPECTS sample, participants completed the CPSS-IV (Foa et al., 2001, p. 17 items), along with three additional items that reflected the new DSM-5 criteria. We used all 20 symptom items from both samples to calculate PTSD severity (see Table S3 for a comparison).

*Harmonisation of scales*. To standardise PTSD symptom scores across studies, UCLA-RI and CPSS-5-SR total scores were rescaled to a 0–1 range by dividing each score by its respective scale maximum.

#### 2.1.3. Self-Reported trauma memory qualities

To assess self-reported trauma memory characteristics, the ASPECTS study utilised the Trauma Memory Quality Questionnaire (TMQQ; Meiser-Stedman et al., 2007), which comprises 11 items assessing the sensory and disorganisation qualities of the trauma memory. The Sinethemba and PROTECT studies administered the Adapted Child Trauma Memory Quality Questionnaire (ATMQQ; Hiller et al., 2021), which includes seven further items. In each dataset, sensory and disorganisation scores were calculated based on the available items, with scoring procedures detailed in Supplementary Table S2. Both sensory and disorganisation scores ranged from 1 to 4. Scores with ≤20% missing values were imputed using the mean of available items.

### 2.2. Data preparation

**Figure 1.**
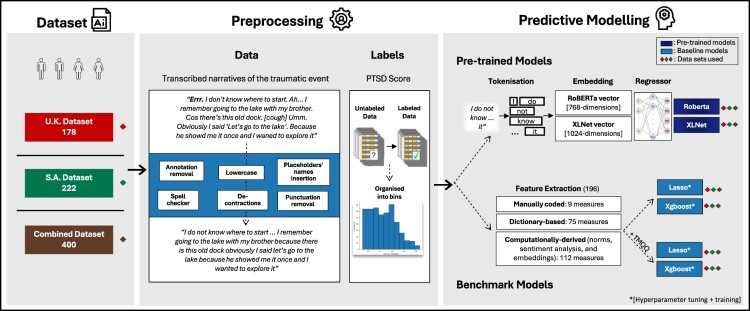
Overview of the datasets and pipelines of the preprocessing and training/finetuning of the predictive models. *UK Dataset*: the dataset from the United Kingdom; *SA Dataset*: the dataset from South Africa.

First, anonymised narratives were pre-processed and labelled with participants’ normalised PTSD scores. Next, two benchmark and two pre-trained models were trained and fine-tuned separately on each dataset and then on the combined dataset. Finally, model performance was evaluated and compared. The complete pipeline (Figure 1) is described in detail in the following sections, and the code is published on GitHub (https://github.com/ag3152/narr_ptsd).

#### 2.2.1. Text preprocessing

A series of text preprocessing steps was applied to standardise the input data. First, anonymised names were replaced with consistent placeholder names, numbers were converted to words, and punctuation, except for question marks, exclamation marks, and periods, was removed to eliminate noise. All text was converted to lowercase for uniformity, contractions were expanded to their full forms, and slang terms were replaced with formal equivalents. Additionally, common misspellings were corrected using the SpellChecker Python package (Barrus, 2025). Stop words (e.g. ‘the,’ ‘to’) were retained. The pre-processed narratives were used to extract the computational features for training the machine learning models.

#### 2.2.2. Labelling

In supervised learning, labelling involves associating input data – trauma narratives – with known outcome values – PTSD severity scores. By training on the labelled examples, the model attempts to identify relationships between narrative characteristics and PTSD severity, allowing it to generate predictions for new, unseen narratives where such associations exist.

#### 2.2.3. Binning

To enable balanced stratification during model training, the scores were initially divided into 10 ascending bins. The number of bins was iteratively adjusted until each contained a minimum threshold of samples, ensuring sufficient representation across all bins. This maintained a proportional distribution of PTSD severity scores in subsequent data splits, reducing the risk of over- or under-representation of specific severity levels in any subset.

#### 2.2.4. Data splits

To fairly evaluate our models, we test them on data that was not used in any part of the training process. Given the limited dataset size, we used 10-fold stratified cross-validation rather than a single train-test split, as it yields more reliable out-of-sample performance estimates. Cross-validation is considered best practice for assessing model generalizability to unseen data when working with small datasets (Wong & Yeh, 2020) and has been used in similar studies (Wshah et al., 2019; Ziobrowski et al., 2021). In this approach, the dataset was randomly partitioned into 10 equal folds, with each iteration using nine folds for training and one fold as a held-out test set. This process was repeated across all folds. Within each fold, the training data were further split into a training subset (80%) and a validation subset (20%), maintaining the proportional distribution of PTSD severity bins through stratification. The training subset was used for model learning, while the validation subset was used for hyperparameter tuning and early stopping to prevent overfitting. All folds were stratified based on class label to preserve the overall class distribution.

### 2.3. Predictive Models

To evaluate whether trauma narratives can predict PTSD severity, we adopted a dual modelling strategy. We compared deep learning models based on LLMs, which learn directly from raw narrative text, and conventional ‘benchmark’ ML models that rely on manually engineered text features. Combining both approaches balances the trade-off between simplicity and interpretability (offered by benchmark models) and LLMs’ potentially higher predictive power and linguistic sensitivity.

#### 2.3.1. Pre-trained large language models

We finetuned two well-established pre-trained large language models: RoBERTa (Liu et al., 2019) and XLNet (Yang et al., 2020). Large language models are deep and unsupervised learning models trained on large text corpora (160 GB of text data for RoBERTa and 130 GB for XLNet) to capture contextual relationships in language. Specifically, both RoBERTa and XLNet have been widely used across various NLP tasks, consistently achieving state-of-the-art or near state-of-the-art performance (Liu et al., 2019; Yang et al., 2020). They have also been successfully applied to narrative-based prediction problems, including sentiment analysis, detection of mental health conditions from text and conversations, and clinical outcome prediction (Chatzimina et al., 2024; Kokane et al., 2024; Shetty et al., 2025).

For this study, pre-processed trauma narratives were tokenised according to each model's vocabulary and converted into numerical vector representations, known as embeddings. RoBERTa generates 768-dimensional embeddings, while XLNet produces 1024-dimensional embeddings, encoding the relationships between words and phrases within the text.

We fine-tuned the RoBERTa-base and XLNet-base-cased models available from HuggingFace (FacebookAI/Roberta-Base · Hugging Face, 2024; Xlnet/Xlnet-Base-Cased · Hugging Face, 2024) by adding a regression layer to output a single continuous PTSD severity score. The fine-tuning process employed a 10-fold stratified cross-validation approach. The models were trained using a mean squared error (MSE) loss function and optimised with the Adam optimiser (learning rate = 1e-6, weight decay = 1e-3, for 25 epochs) (Kingma & Ba, 2017). The best-performing epoch was selected based on the lowest validation loss, and the final model performance was evaluated using Root Mean Squared Error (RMSE) and Mean Absolute Error (MAE) metrics. RMSE measures the average magnitude of prediction errors by penalising larger errors more heavily, while MAE represents the average absolute difference between predicted and actual values, both commonly used to assess regression model performance (Chai & Draxler, 2014). Additionally, we computed the *R*² score to measure explained variance and Pearson’s correlation to assess the strength of the associations between predicted and actual PTSD scores. To address class imbalance in PTSD severity scores, weighted random sampling assigned higher probabilities to underrepresented severity levels by setting weights inversely proportional to class frequencies.

#### 2.3.2. Benchmark models

*Feature Extraction*. To train the benchmark models, we extracted a set of interpretable features from participants’ trauma narratives, guided by prior empirical and theoretical work linking specific narrative characteristics to PTSD symptoms in youth. These features broadly capture four dimensions: coherence and disorganisation, sensory-perceptual language, emotional valence and arousal, and linguistic style and complexity. Feature selection was informed by a synthesis of existing literature on narrative markers of PTSD (e.g.; Crespo & Fernández-Lansac, 2016; O’Kearney & Perrott, 2006; Rubin et al., 2008, 2016), as well as theoretical models of trauma memory (e.g. Brewin, 2014; Meiser-Stedman et al., 2007). The dataset was then standardised using z-score normalisation to ensure comparability across features. A full list of features, operational definitions, and extraction methods is provided in Table S1.

*Lasso, XGBoost.* We implemented two widely used machine learning models that capture linear and non-linear relationships, respectively, LASSO regression and XGBoost. PTSD severity scores served as the outcome variable. A random search was conducted over predefined hyperparameter grids to optimise model performance. As for the pre-trained approach, model performance was assessed using RMSE, MAE, *R*² score and Pearson correlation between predicted and actual PTSD scores.

To validate our approach, we tested whether the inclusion of self-reported memory characteristics – proven predictors of PTSD development (McGuire et al., 2021) – enhanced predictive performance. Specifically, we incorporated sensory and disorganisation scores from the TMQQ into the feature set and repeated the training procedure. The hypothesis was that these external, theoretically grounded predictors would improve model performance, thereby validating our linguistic and embedding-based feature extraction approach.

Finally, to determine the standalone predictive value of the TMQQ subscales, we will conduct an ablation approach, training the same models using only the TMQQ scores as input features.

## 3. Results

### 3.1. Descriptive statistics

The study sample consisted of 400 participants aged 6–18 years (*M* = 11.81, SD = 2.76). A variety of traumatic experiences were represented, and the distributions of PTSD symptom severity, narrative lengths, and TMQQ scores are summarised in Table 1. PTSD scores were positively skewed in both samples, and narrative lengths differed between the UK and South African samples.

**Figure 2.**
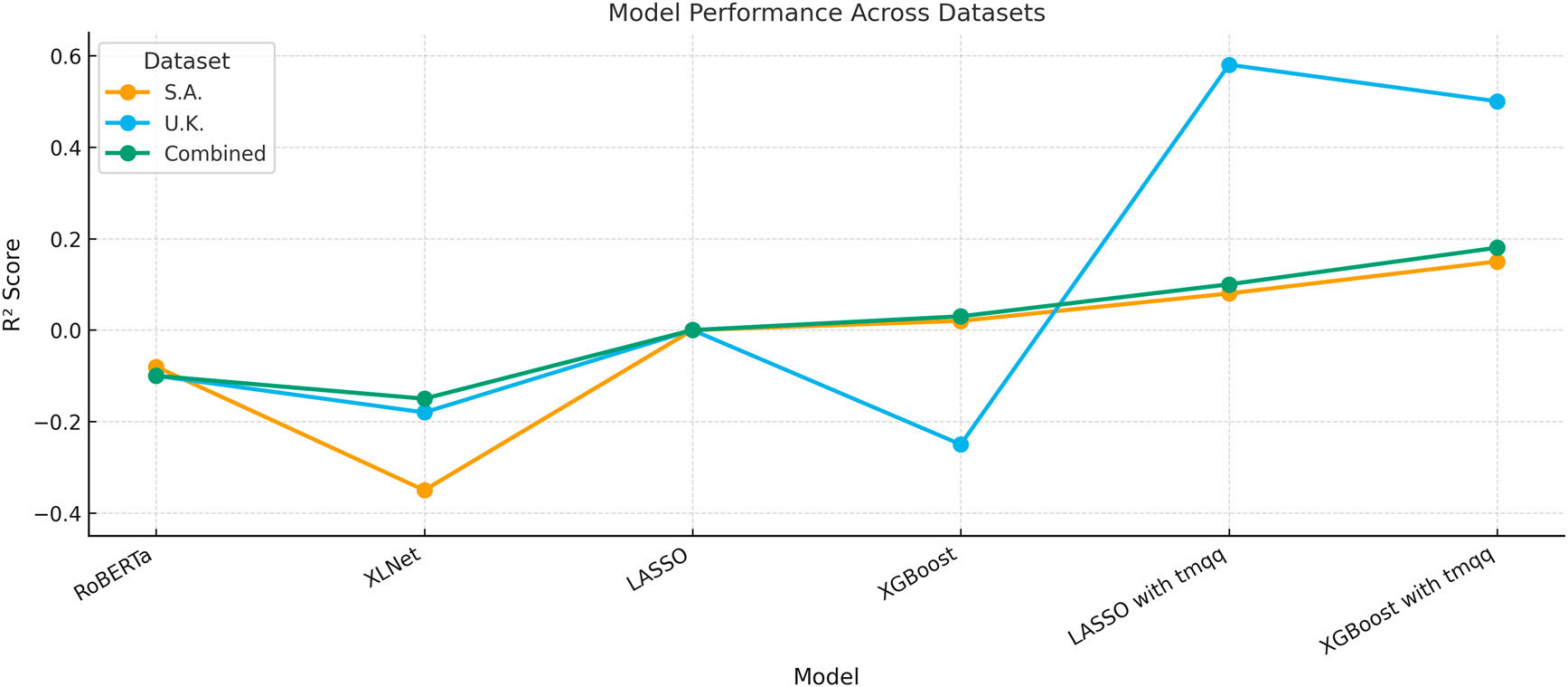
*R*^2^ scores for PTSD prediction across models and datasets.

**Figure 3.**
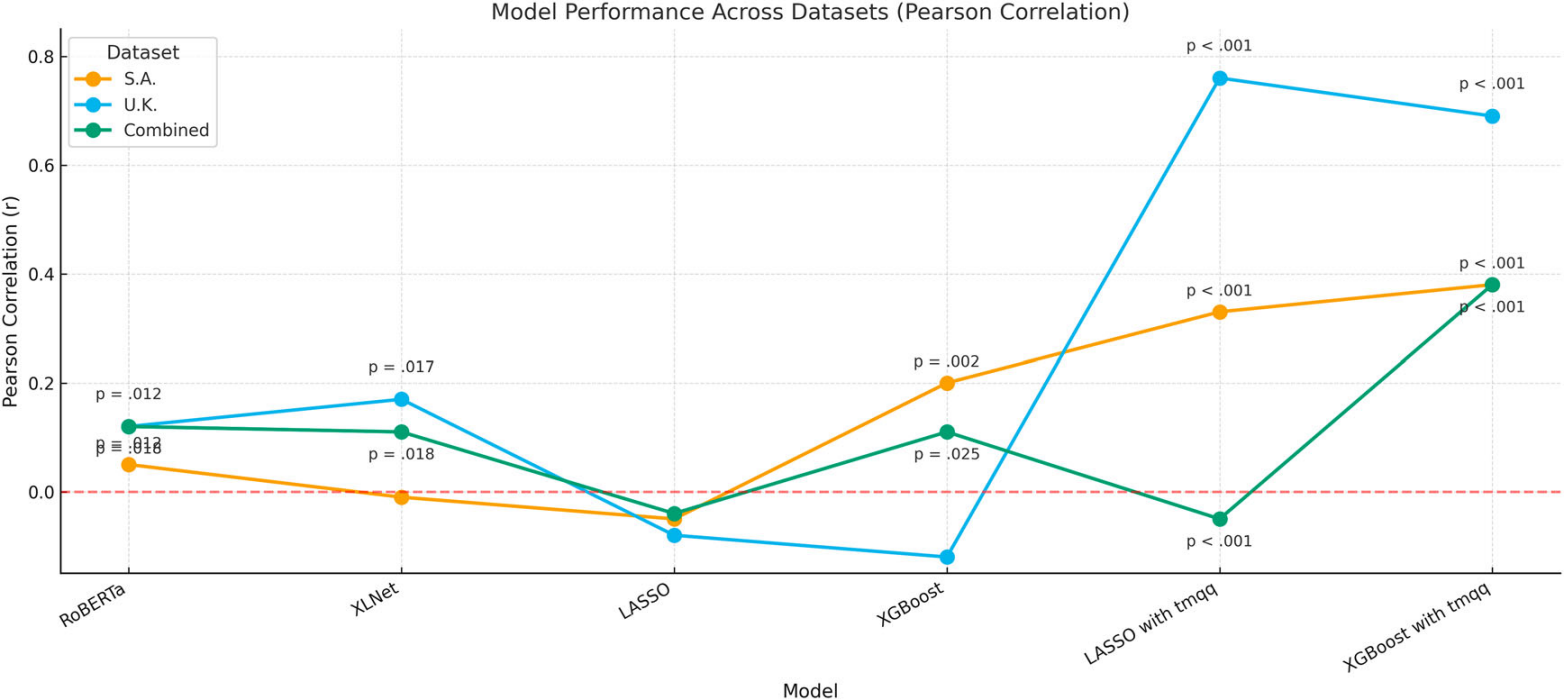
Pearson correlation coefficients between predicted and actual PTSD scores across models and datasets. Only *p*-values < .05 are shown.

**Table 1.** Descriptive statistics for the sample demographics, trauma types, PTSD severity scores, narrative word counts, and TMQQ scores.

Measures	UK Dataset (*N* = 178)	SA Dataset (*N* = 222)	Combined Dataset (*N* = 400)
**Age**	*M* = 10.91 (SD = 2.77) Range: 6–17.74	*M* = 12.55 (SD = 2.53) Range: 8.04–16.95	*M* = 11.81 (SD = 2.76) Range: 6–17.74
**Traumatic Event**			
Road Traffic Accident	88	55	143
Accidental Injury	56	87	143
Assault	12	32	44
Acute Medical Emergency	10	1	11
Dog Bite	4	0	4
Other	14	41	55
**Trauma Narrative Word Count**	*M* = 422 (SD = 351) Range: 26–1841	*M* = 263 (SD = 297), Range: 19–2262	*M* = 336 (SD = 332), Range: 19–2262
**Raw PTSD scores**	^A^M = 19.91 (SD = 17.18) Range: 0–80 ^P^M = 21.21 (SD = 14.99) Range: 0–60	*M* = 25.94 (SD = 13.18) Range: 0–60	
**Harmonised PTSD scores** (0-1 range)	*M* = 0.28 (SD = 0.23)	*M* = 0.32 (SD = 0.16)	*M* = 0.30 (SD = 0.20)
**TMQQ-sensory scores** (0-4 range)	*M* = 2.25 (SD = 0.86)	*M* = 2.35 (SD = 0.65)	*M* = 2.25 (SD = 0.67)
**TMQQ-disorganisation scores** (0-4 range)	*M* = 2.12 (SD = 0.68)	*M* = 2.42 (SD = 0.59)	*M* = 2.34 (SD = 0.72)

Note: A = ASPECT, P = PROTECT.

### 3.2. Predictive utility of narratives using LLMs

First, we employed two state-of-the-art LLMs (RoBERTa and XLNet) fine-tuned to learn contextual representations directly from raw text, to test the capacity of these approaches to predict PTSD symptom scores from children’s trauma narratives. Fine-tuning on children’s trauma narratives failed to produce meaningful predictions across datasets (Figure 2). In the combined dataset, neither RoBERTa nor XLNet explained any variance in PTSD severity, and both yielded negative *R*² values (RoBERTa *R*²  =  –.05; XLNet *R*²  =  –.23), indicating that their predictions were worse than using the sample mean as a baseline. While weak but statistically significant correlations were observed between predicted and actual PTSD scores (*r* ≈ .12), these are likely artefacts of sample size and not indicative of meaningful predictive power. Similar results emerged in the dataset-specific analyses, with neither model accounting for variance in the UK or SA samples. Detailed performance metrics are presented in Table 2.

**Table 2.** Pre-trained large language model performances trained on narratives based on the RMSE, MAE, *R*^2^ metrics and Pearson correlation between predicted and actual PTSD scores.

Dataset	Model	RMSE	MAE	*R*^2^	Pearson *r* (*p*-value)
UK	RoBERTa	0.24	0.20	−0.09	0.13 (*p* = .08)
	XLNet	0.25	0.21	−0.18	0.17 (*p* = .02)
SA	RoBERTa	0.17	0.13	−0.04	0.05 (*p* = .49)
	XLNet	0.19	0.15	−0.39	−0.008 (*p* = .9)
Combined	RoBERTa	0.20	0.17	−0.05	0.12 (*p* = .01)
	XLNet	0.22	0.18	−0.23	0.12 (*p* = .02)

### 3.3. predictive utility of narrative features using benchmark models

Second, we used two standard benchmark algorithms – linear LASSO regression and XGBoost – to test whether already manually extracted narrative features could predict children’s PTSD. Full performance metrics and correlation coefficients between actual and predicted PTSD scored are presented in Table 3 and FIgure 3. In the combined dataset, both models performed at a chance level: LASSO explained no variance (*R*² ≈ 0, RMSE  =  0.19), and the correlation between predicted and observed PTSD scores was negligible (*r*  =  –.04). XGBoost performed similarly, with an *R*² of –.01 and an RMSE of 0.20. The UK sample yielded similar results, with non-significant Pearson correlations for both models. In the South African sample, XGBoost produced a modest correlation between predicted and actual PTSD scores (*r*  =  .21, *p*  =  .001), contrary to LASSO. Still, the variance explained remained minimal for both models.

### 3.4. Incremental contribution of TMQQ scores

Finally, we tested whether adding children’s self-reported trauma memory characteristics – sensory and disorganisation scores from the TMQQ – would improve the prediction of PTSD beyond using narrative features alone. Full performance metrics are reported in Table 3. In the combined dataset, model performance did not improve for LASSO, which explained minimal variance and produced a near-zero correlation. In contrast, XGBoost showed better outcomes, with *R*²  =  .14 and *r*  =  .37 (*p* < .001), outperforming the model when trained only on narrative features.

Performance gains were more substantial within each dataset. In the UK sample, LASSO’s predictive power increased sharply: *R*² rose to .57 and *r* reached .75 (*p* < .001). XGBoost improved similarly (*R*²  =  .45, *r*  =  .67, *p* < .001). In the South African sample, improvements were smaller but still significant: LASSO reached *R*²  =  .09 and *r*  =  .32 (*p* < .001), while XGBoost performed slightly better (*R*²  =  .14, *r*  =  .38, *p* < .001).

Supplementary Figure S5-7 presents feature importance plots for all these models.

Finally, when used as the sole input features to train the models, self-reported memory characteristics yielded stronger predictive performance than when combined with extracted narrative features, particularly in the UK and combined datasets, as shown in the final section of Table 3 and in Figure 3.

**Table 3.** Performance of benchmark models trained on features extracted from trauma narratives alone, on features extracted from trauma narratives along with TMQQ sensory and disorganisation self-reported scores, and on TMQQ sensory and disorganisation self-reported scores. Models’ performances are evaluated using RMSE, MAE, *R*², and Pearson correlation between predicted and actual PTSD scores.

Dataset	Model	RMSE	MAE	*R*^2^	Pearson *r* (*p* value)
**UK**	Lasso	0.23	0.19	−0.001	−0.08 (*p* = .3)
** **	XGBoost	0.23	0.19	−0.06	0.11 (*p* = .1)
**SA**	Lasso	0.16	0.13	−0.001	−0.08 (*p* = .3)
** **	XGBoost	0.16	0.13	0.007	0.21 (*p* = .001)
**Combined**	Lasso	0.19	0.16	−0.0004	−0.04 (*p* = .4)
** **	XGBoost	0.20	0.16	−0.01	0.11 (*p* = .03)
*Adding TMQQ sensory and disorganisation scores*					
**UK**	Lasso	0.15	0.11	0.57	0.75 (*p* < .001)
** **	XGBoost	0.17	0.13	0.45	0.67 (*p* < .001)
**SA**	Lasso	0.16	0.12	0.09	0.32 (*p* < .001)
** **	XGBoost	0.15	0.12	0.14	0.38 (*p* < .001)
**Combined**	Lasso	0.19	0.16	−0.0004	−0.04 (*p* = .4)
** **	XGBoost	0.18	0.15	0.14	0.37 (*p* < .001)
*Using only TMQQ sensory and disorganisation scores as input features*					
**UK**	Lasso	0.14	0.11	0.58	0.76 (*p* < .001)
** **	XGBoost	0.16	0.12	0.53	0.73 (*p* < .001)
**SA**	Lasso	0.15	0.12	0.11	0.33 (*p* < .001)
** **	XGBoost	0.16	0.12	0.11	0.33 (*p* < .001)
**Combined**	Lasso	0.19	0.16	0.02	0.17 (*p* < .001)
** **	XGBoost	0.18	0.15	0.13	0.37 (*p* < .001)

## 4. Discussion

This study is the first to apply machine learning to children and adolescents’ trauma narratives to evaluate whether linguistic features can predict post-traumatic stress symptom severity. We further investigated whether these predictions generalise across cultural contexts (UK and South Africa) and whether incorporating subjective trauma memory appraisals improves model performance. We aimed to evaluate the feasibility of scalable, low-cost tools for PTSD risk identification in youth, an area of growing relevance.

We found that trauma narratives alone consistently failed to yield meaningful predictions of PTSD severity across datasets and modelling approaches. This null result contrasts prior research in adult samples, where linguistic feature-based ML approaches have been used to detect several psychological disorders, including PTSD, depression, anxiety and bipolar disorders, with moderate-to-high accuracy (Calvo et al., 2017; Coppersmith et al., 2014b; Voleti et al., 2019; Zhang et al., 2022). However, these studies did not rely on oral, autobiographical narratives, but rather on structured clinical data or social media posts. These written or highly structured texts are more deliberate and lexically denser, properties that generally benefit text-only NLP models. Moreover, oral, autobiographical accounts lose prosodic and paralinguistic information when transcribed (e.g. pitch variability, timbre, intensity) that carry affective signal and have shown promise for PTSD detection in speech-based work (Marmar et al., 2019; Quatieri et al., 2023). One exception is Bartal et al.’s (2024) study that used ML to detect post-partum PTSD from childbirth narratives with promising results, but their work involved written, rather than spoken accounts.

While no other study has directly applied ML to oral trauma narratives, studies using manually coded linguistic features have identified distinct patterns in trauma recollection, at least among adults. A systematic review found that adults with PTSD tend to recall traumatic events with heightened sensory, perceptual, and emotional detail (Crespo & Fernández-Lansac, 2016). In contrast, those with fewer PTSD symptoms describe their experiences in a more neutral or less distressing manner (Dekel & Bonanno, 2013). By contrast, our findings suggest that PTSD symptomatology in young individuals may not manifest in trauma narratives in a way that is reliably detectable by ML models, despite the inclusion of a comprehensive set of theory-driven linguistic features, capturing memory fragmentation, emotional intensity, narrative coherence, and other markers previously associated with PTSD. We also failed to establish reliable PTSD prediction using state-of-the-art LLMs, which can capture complex associations between language and psychological outcomes, including features that are difficult to formalise or manually extract.

It is notable that, compared to the adult field, evidence relating to the predictive value of manually coded trauma narrative among young individuals is also less consistent. The ongoing context of linguistic development may present challenges to extracting clinically meaningful narrative features among children and adolescents. Children and adolescents exhibit greater variability in memory representations and expressive capacities due to ongoing cognitive, emotional, and linguistic development (Fivush, 2011; Nelson & Fivush, 2004). As a result, their trauma narratives are likely to be fundamentally more variable in terms of key features such as sensory and emotional expression and linguistic coherence, independent of clinical status, making it difficult for ML models to discern signal from noise. Another possibility is that the narratives contain fewer clinical signals because avoidance, a core symptom of the disorder (APA, 2013), may lead children and adolescents to omit distressing aspects of the trauma, thereby limiting the content available for predictive modelling. Future research should explore whether similar ML approaches yield stronger results when applied to trauma narratives from adults, to clarify whether our findings reflect a developmental limitation or a more general limitation of language-based models in PTSD predictions. Moreover, since assessments included in this study were conducted around the DSM-5 one-month diagnostic threshold (American Psychiatric Association, 2013), some presentations may have reflected acute stress rather than more stable symptom profiles. Future work should assess whether narratives obtained later yield a stronger predictive signal for diagnosed PTSD.

A second key finding was that self-reported trauma memory characteristics were more suitable predictors of PTSD symptom severity than narrative features in this study. We caution, however, that part of this advantage may reflect shared method variance: both memory appraisals and symptom severity are self-reported, potentially inflating associations via overlapping channels of emotional expression. At the same time, our result aligns with prior findings suggesting that subjective memory appraisals, such as perceptions of fragmentation, vividness, or coherence, are closely related to PTSD severity (Bryant et al., 2007; Rubin et al., 2016). Our results reinforce cognitive models of PTSD (Ehlers & Clark, 2000), which emphasise the role of maladaptive memory representations in symptom maintenance, suggesting that how an individual internally appraises their trauma memory is more predictive than narrative content or structure. Taken together, in this sample of children and adolescents, self-reported trauma memory qualities outperformed narrative-derived features for predicting PTSD symptom severity. This may be because self-reports directly index core PTSD phenomenology (perceived disorganisation, sensory vividness, intrusiveness), capturing underlying memory processes that narratives may fail to convey, especially in children and adolescents due to developing language skills and expressive capacities. Future work should test whether this pattern generalises across samples, languages, and alternative methods for extracting and analysing narrative features.

The greater predictive accuracy observed in the UK sample could reflect contextual differences in self-report competencies, as opportunities for using metacognitive vocabulary and norms for describing internal states vary cross-culturally (Wang et al., 2010). It could also be caused by differences in instrument validity across sites. Even with careful translation and adaptation, subtle shifts in item semantics or response styles can alter effective score range, yielding stronger prediction in the UK. Future work should establish measurement invariance and consider culturally adapted instruments to clarify whether the gap reflects true differences or measurement/context effects.

Our results carry both theoretical and clinical implications. First, they challenge the assumption that trauma narratives can serve as reliable indicators of PTSD severity among young individuals. Although cognitive models of PTSD suggest that memory characteristics such as fragmentation and disorganisation are linked to symptomatology (Ehlers & Clark, 2000; Foa et al., 1995), our findings indicate that these characteristics may not be consistently externalised in trauma narratives in a way that machine learning models can detect, at least in children and adolescents. This adds a critical layer to current cognitive models of PTSD, emphasising that the subjective experience of memory, rather than its linguistic manifestation, may be the most clinically relevant feature. From a practical standpoint, our results suggest that research efforts among trauma-exposed young individuals should not focus on developing predictive models based solely on trauma narratives. Such models demonstrated little predictive power and require substantial computational resources. Instead, subjective memory characteristics, as well as other easily collectable variables such as demographic or psychological assessments, may be a more viable focus for future ML models.

### 4.1. Limitations and future directions

Despite our robust study design, some key limitations must be acknowledged. First, in ML, dataset size critically influences a model’s capacity to discern underlying patterns, with larger datasets enhancing generalizability and predictive performance. While the current sample is large for the PTSD field, it is modest relative to large-scale natural language processing tasks, which may limit the ability to capture subtle linguistic patterns associated with PTSD severity. Second, we relied on text-based analysis, which does not capture prosodic and paralinguistic cues (e.g. tone, pitch, and rhythm). Preliminary evidence indicates that ML analysis based on acoustic features of speech may distinguish adults with and without PTSD (Hu et al., 2024), and ensemble methods that integrate text and speech data have also shown improved performance in predicting mental health disorders (Mao et al., 2023). Future research should integrate speech-based analyses to determine whether PTSD is reflected in acoustic markers in children and adolescents. Third, our study was based on data derived from across the UK and South Africa. While this can enhance the generalisability of our findings, it also introduces heterogeneity. Fourth, limited item-level missingness was addressed using within-person proration to preserve each child’s score profile. However, this approach can attenuate variance or bias estimates; future work should consider multiple imputation, likelihood-based approaches, or ML-based imputation techniques to more rigorously handle item-level missing data. Finally, pooling data across different studies required harmonising two different PTSD instruments. While essential to increase the dataset, this standardisation may have obscured instrument-specific scaling and psychometric differences, potentially diluting the signal. In addition, future work might collect a neutral or positive narrative for each participant; comparing the trauma account to this baseline could help disentangle trauma-specific changes from stable individual or cultural differences in expression, thereby clarifying the predictive signal.

### 4.2. Conclusions

Our findings suggest that, under the datasets, features, and modelling approaches used here, trauma narratives alone did not yield reliable prediction of PTSD severity in children and adolescents. By contrast, self-reported trauma memory characteristics was a more suitable predictor of PTSD severity, suggesting that subjective memory appraisals are more closely linked to PTSD severity than linguistic expression in young individuals. These results carry both theoretical and clinical significance. Theoretically, they challenge assumptions that trauma memory disorganisation is consistently observable in narrative accounts, at least among children and adolescents. Clinically, they suggest that efficient, scalable PTSD screening in youth may benefit more from brief self-report tools than from narrative analysis, particularly in low-resource or high-burden settings. Future research should prioritise clinically relevant, accessible, and theoretically grounded features, such as self-reported trauma memory appraisals, to enhance early identification and intervention for PTSD in youth.

## Supplementary Material

Supplemental Material

## Data Availability

The data analysed in this study are confidential. To request access, please contact slh54@bath.ac.uk and R.Meiser-Stedman@uea.ac.uk for individual datasets.
