# Supplementary material for: Can machine learning predict PTSD symptoms from trauma narratives of children and adolescents?
Source: Eur J Psychotraumatol. 2025 Dec 2;16(1):2589709. doi: 10.1080/20008066.2025.2589709 (PMC12673981; doi:10.1080/20008066.2025.2589709)

**Supplementary Material**

**Table S1.** Features extracted from the narratives are used as inputs for training the benchmark models. The features are categorised and include their respective extraction methods. Manual scores were standardised into z-scores to account for differences in verbosity. In the word count-based approach, scores represent the percentage of words belonging to specific categories relative to the total text length.

| **Category** | **Features** | **Extraction Method** |
| --- | --- | --- |
| ***Manual Coding*:***  **Emotions**  **Sensory Information**  **Disorganisation** | negative emotions, positive emotions  pain utterances  organised thoughts, disorg thoughts, repetitions | Using Foa et al. (1995)’s manual scheme |
| ***Sentiment Analysis*** | Vader score, Flair score | Extracted using Vader (Hutto, 2014/2025) and Flair (*flairNLP/Flair*, 2018/2025) packages |
| ***Linguistic Features*** | concreteness, valence, arousal, Analytic, Clout, Authentic, Tone, WPS, Sixltr, Dic, function | Extracted using LIWC (Pennebaker et al., 2015), concreteness (Brysbaert et al., 2014) and valence norms (Warriner et al., 2013) in R |
| ***Text Cohesion*** | word2vec_1_all_sent, word2vec_2_all_sent, lsa_1_all_sent, lsa_2_all_sent, lda_1_all_sent, lda_2_all_sent | Extracted using Glove* (Crossley et al., 2016) |
| ***Word Count-based:***  **Emotions**  **Sensations**  **Cognitive Processing**  **Personal Pronouns**  **Time Focus**  **Space**  **Social Connections**  **Communication Style**  **Parts of Speech**  **Word Frequencies** | posemo, negemo, anx, anger, sad  percept, see, hear, feel  cogproc, insight, cause, discrep, tentat, certain,  pronoun, ppron, i, we, you, shehe, they, ipron  focuspast, focuspresent, focusfuture, time  relativ, motion, space, time  social, family, friend, female, male  informal, swear, netspeak, assent, nonflu, filler  article, prep, auxverb, adverb, conj, negate, verb, adj, compare, interrog  bio, body, health, sexual, ingest, drives, affiliation, achieve, power, reward, risk, work, leisure, home, money, relig, death | Extracted using LIWC (Pennebaker et al., 2015) |
| ***Lexical Analysis*** | lemma_mattr, lexical_density_tokens, lexical_density_types, all *_tr, all adjacent_overlap_*, syn_overlap_sent_noun, syn_overlap_sent_verb,, basic_connectives, conjunctions, disjunctions, lexical_subordinators, coordinating_conjuncts, addition sentence_linking order, reason_and_purpose, all_causal, positive_causal, opposition, determiners, all_demonstratives, attended_demonstratives, unattended_demonstratives, all_additive, all_logical, positive_logical, negative_logical, all_temporal, positive_intentional, all_positive, all_negative, all_connective, pronoun_density, pronoun_noun_ratio, repeated_content_lemmas, repeated_content_and_pronoun_lemmas | Extracted using LFTK** (Lee & Lee, 2023) |

*For GloVe, word embeddings were derived from the GloVe 6B (50-dimensional) vectors. Each narrative was tokenised using spaCy, and a mean embedding was computed for each participant. **LFTK stands for Linguistic Feature Toolkit, a Python package which computes a broad set of syntactic and lexical characteristics from text.

**Table S2.** CPSS Symptom Items Across Studies. The items follow the prompt “Read each question carefully. Then circle the number (0-4) that best describes how often that problem has bothered you IN THE LAST MONTH.”

| **Item No.** | **CPSS-IV with DSM-5-Aligned**  **Additions (ASPECTS)** | | **CPSS-5-SR (Sinethemba)** |
| --- | --- | --- | --- |
| 1 | Having upsetting thoughts or images about the event that came into your head when you didn’t want them to | Having upsetting thoughts or pictures about it that came into your head when you didn’t want them to | |
| 2 | Having bad dreams or nightmares | Having bad dreams or nightmares | |
| 3 | Acting or feeling as if it was happening again (seeing or hearing something and feeling as if you are there again) | Acting or feeling as if it was happening again (seeing or hearing something and feeling as if you are there again) | |
| 4 | Feeling upset when you think about it or hear about the event (for example, feeling scared, angry, sad, guilty, confused) | Feeling upset when you think about it or hear about the event (for example, feeling scared, angry, sad, guilty, confused) | |
| 5 | Having feelings in your body when you think about or hear about the event (for example, breaking out into a sweat, heart beating fast) | Having physical reactions when reminded of the event (for example, sweating, heart beating fast, stomach or head hurting) | |
| 6 | Trying not to think about, talk about, or have feelings about the event | Trying not to think about it or have feelings about it | |
| 7 | Trying to avoid activities, people, or places that remind you of the traumatic event | Trying to stay away from anything that reminds you of what happened (for example, people, places, or conversations about it) | |
| 8 | Not being able to remember an important part of the upsetting event | Not being able to remember an important part of what happened | |
| 9 | Having much less interest or doing things you used to do | Having bad thoughts about yourself, other people, or the world (for example, “I can’t do anything right”, “All people are bad”, “The world is a scary place”) | |
| 10 | Not feeling close to people around you | Thinking that what happened is your fault (for example, “I should have known better”, “I shouldn’t have done that”, “I deserved it”) | |
| 11 | Not being able to have strong feelings (for example, being unable to cry or unable to feel happy) | Having strong bad feelings (like fear, anger, guilt, or shame) | |
| 12 | Feeling as if your future plans or hopes will not come true (for example, you will not have a job or getting married or having kids) | Having much less interest in doing things you used to do | |
| 13 | Having trouble falling or staying asleep | Not feeling close to your friends or family or not wanting to be around them | |
| 14 | Feeling irritable or having fits of anger | Trouble having good feelings (like happiness or love) or trouble having any feelings at all | |
| 15 | Having trouble concentrating (for example, losing track of a story on the television, forgetting what you read, not paying attention in class) | Getting angry easily (for example, yelling, hitting others, throwing things) | |
| 16 | Being overly careful (for example, checking to see who is around you and what is around you) | Doing things that might hurt yourself (for example, taking drugs, drinking alcohol, running away, cutting) | |
| 17 | Being jumpy or easily startled (for example, when someone walks up behind you) | Being very careful or on the lookout for danger (for example, checking to see who is around you and what is around you) | |
| 18 | Completely blaming myself or someone else for what happened. | Feeling very negative (e.g., fear, anger, guilt, or shame) | |
| 19 | Feeling scared, angry, guilty or ashamed a lot of the time. | Having trouble paying attention (for example, losing track of a story on TV, forgetting what you read, unable to pay attention in class) | |
| 20 | Thinking that the world is very dangerous or that your life has been ruined by what happened. | Having trouble falling or staying asleep | |

**Note**: Items 18–20 reflect additional DSM-5 symptom criteria included in the CPSS-5-SR. In ASPECTS, these were administered as supplementary items alongside the CPSS-IV to enable DSM-5-aligned scoring.

**Table S3.** Item content and subscale mapping for the Trauma Memory Quality Questionnaire (TMQQ) and Adapted Child Trauma Memory Quality Questionnaire (ATMQQ). The first 11 items were common to both the TMQQ (used in the ASPECTS study) and the ATMQQ (used in the Sinethemba and PROTECT studies). The ATMQQ additionally included seven items (items 12–18). Sensory and disorganisation scores were derived from the relevant items available in each dataset.

| **Item No.** | **Item Text** | **Subscale** |
| --- | --- | --- |
| 1 | My memories of the frightening event are mostly pictures or images. | Sensory |
| 2 | I can’t seem to put the frightening event into words. | Disorganisation |
| 3 | When I have memories of what happened I sometimes hear things in my head that I heard during the event. | Sensory |
| 4 | When I remember the frightening event I feel like it is happening right now. | Sensory |
| 5 | When I think about the frightening event I can sometimes smell things that I smelt when it happened. | Sensory |
| 6 | I can talk about what happened very easily. | Disorganisation |
| 7 | I remember the frightening event as a few moments, and each moment is a picture in my mind. | Sensory |
| 8 | My memories of the frightening event are like a film that plays over and over. | Sensory |
| 9 | My memories of the frightening event are very clear and detailed. | Sensory |
| 10 | Remembering what happened during the frightening event is just like looking at photographs in my mind. | Sensory |
| 11 | When memories come to mind of what happened, I feel my body is in the same position as during the event. | Sensory |
| 12 | I get mixed up about what order things happened in during the frightening event. | Disorganisation |
| 13 | There are some parts of the frightening event that I can’t really remember. | Disorganisation |
| 14 | I don’t have a complete story about what happened during the frightening event. | Disorganisation |
| 15 | My memory of the frightening event is muddled. | Disorganisation |
| 16 | I don’t think I’ve remembered everything about the frightening event. | Disorganisation |
| 17 | It is hard for me to sort out what I remember about the frightening event. | Disorganisation |
| 18 | When I remember what happened I forget where I am right now. | Disorganisation |

**Figure S1.** Scatter plots of predicted vs. actual PTSD severity scores for pre-trained models fine-tuned on trauma narratives across all datasets. The alignment of points along the diagonal reflects the accuracy of the model’s predictions, with tighter clustering along the line indicating stronger predictive performance.

| **Dataset** | **RoBERTa** | **XLNet** |
| --- | --- | --- |
| **Combined** | 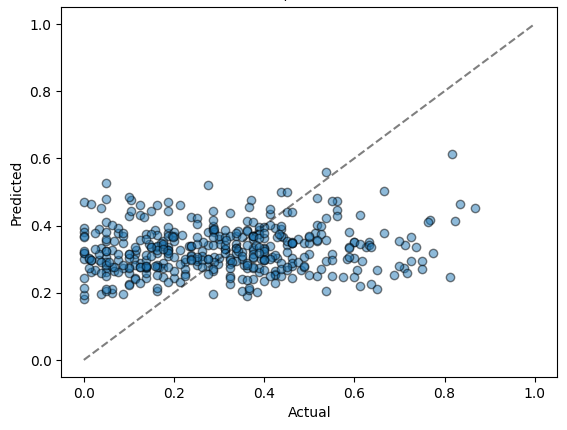 | 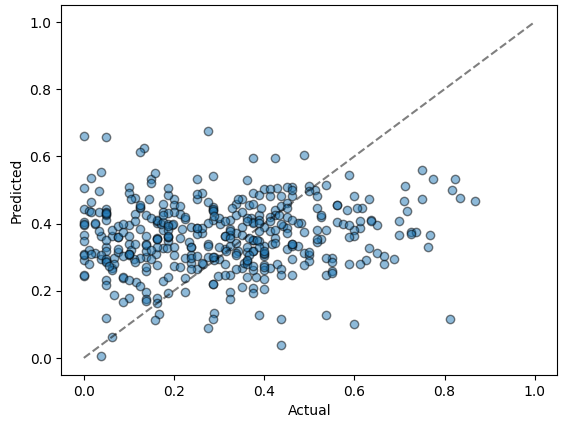 |
| **U.K.** | 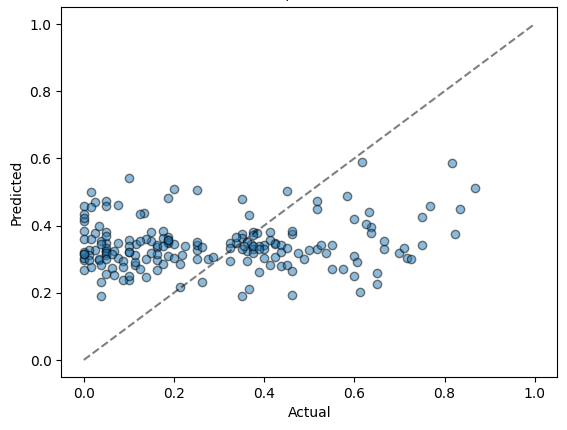 | 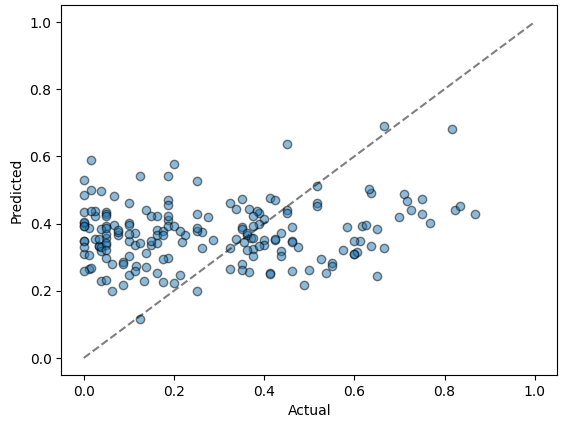 |
| **S.A.** | 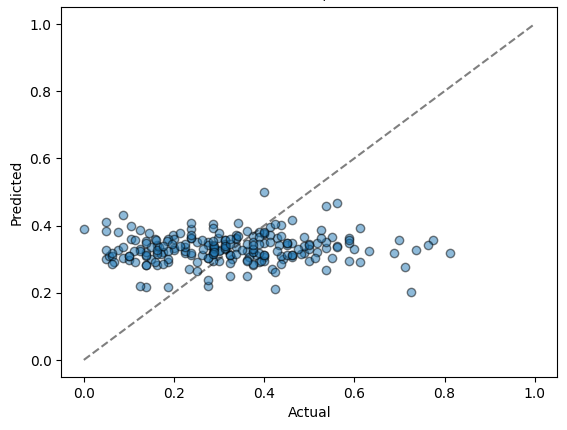 | 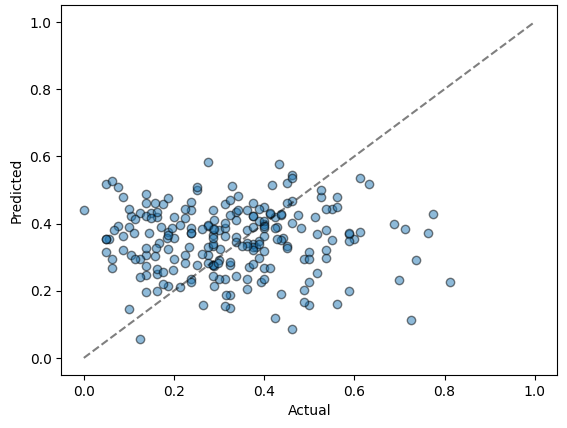 |

**Figure S2.** Scatter plots of predicted vs. actual PTSD severity scores for benchmark models (LASSO and XGBoost) trained on trauma narratives alone across all datasets. The dispersion of points away from the diagonal suggests weaker predictive accuracy when using linguistic features in isolation.

| **Dataset** | **Lasso** | **XGBoost** |
| --- | --- | --- |
| **Combined** | 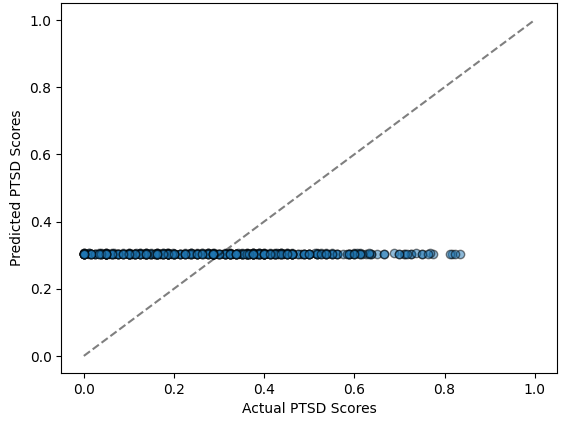 | 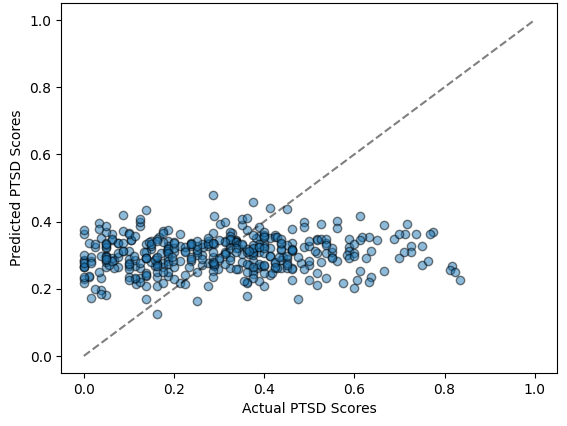 |
| **U.K.** | 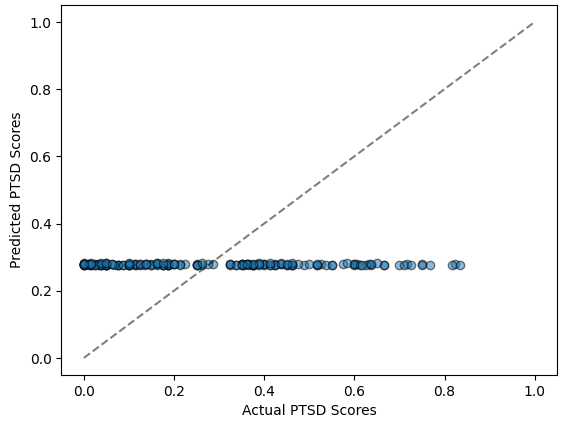 | 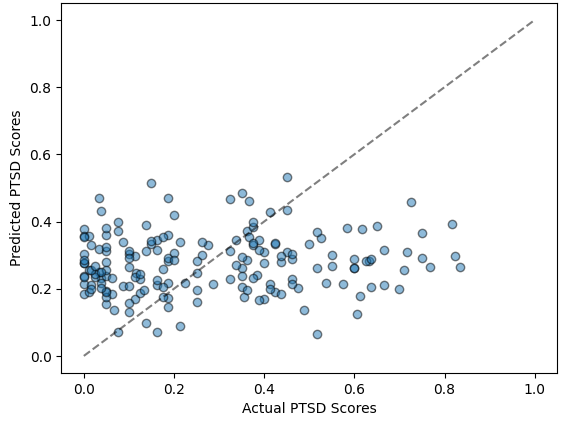 |
| **S.A.** | 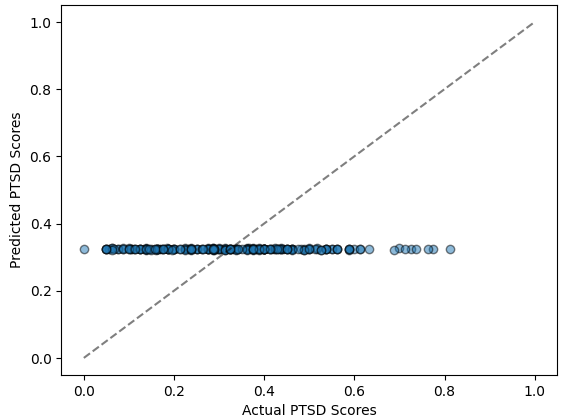 | 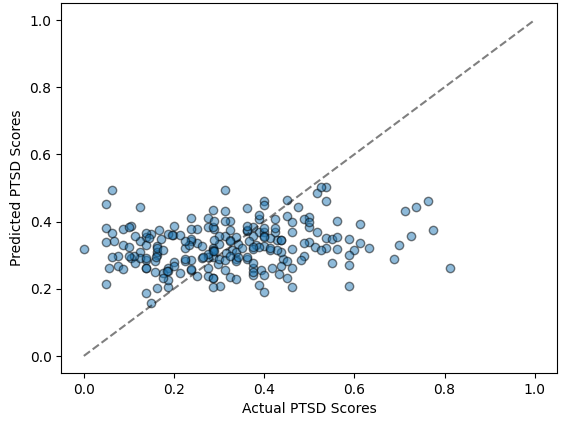 |

**Figure S3.** Scatter plots of predicted vs. actual PTSD severity scores for benchmark models (LASSO and XGBoost) trained on trauma narratives and TMQQ sensory and disorganisation self-reported scores across all datasets.

| **Dataset** | **Lasso** | **XGBoost** |
| --- | --- | --- |
| **Combined** | 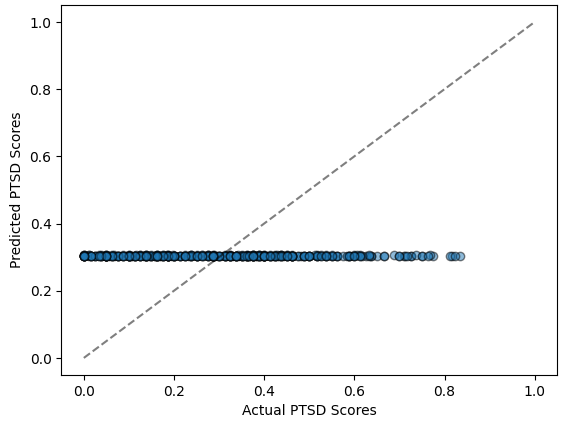 | 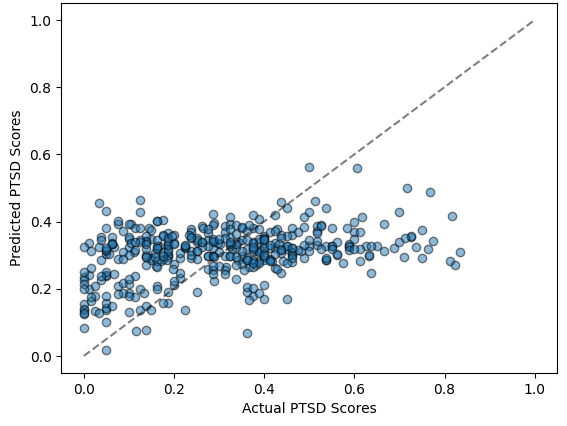 |
| **U.K.** | 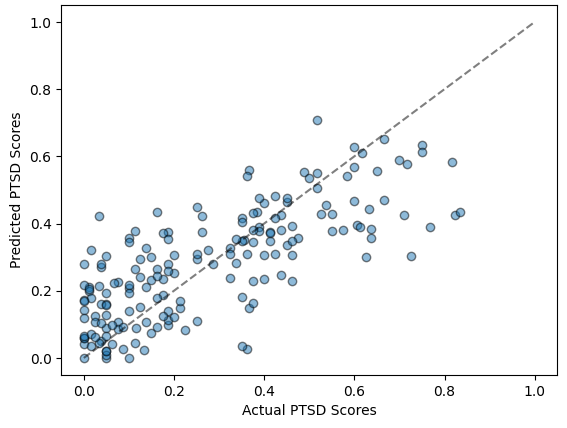 | 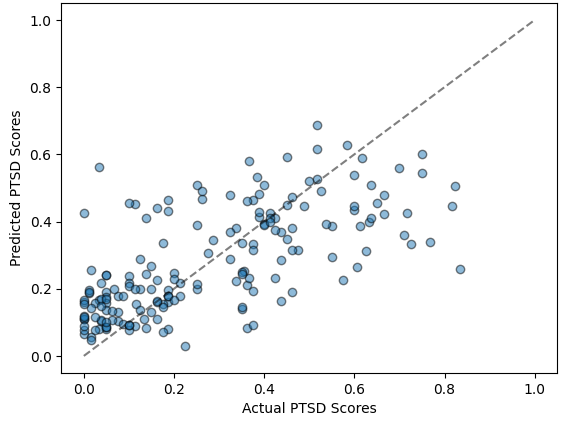 |
| **S.A.** | 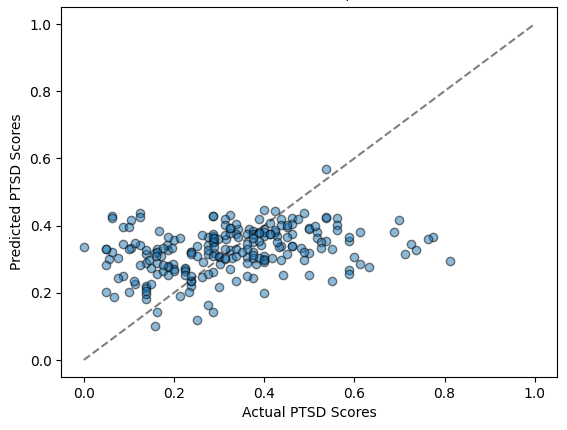 | 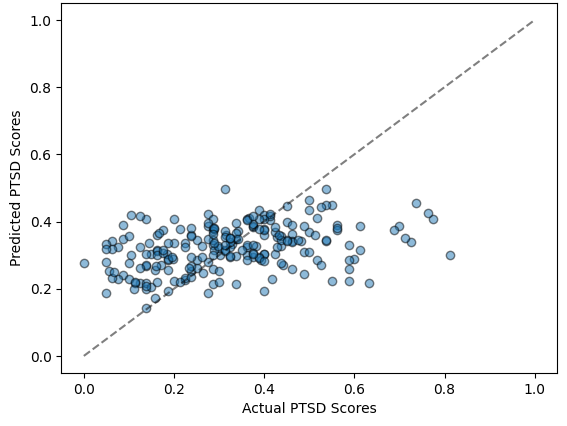 |

**Figure S4.** Scatter plots of predicted vs. actual PTSD severity scores for benchmark models (LASSO and XGBoost) trained only on TMQQ sensory and disorganisation self-reported scores across all datasets.

| **Dataset** | **Lasso** | **XGBoost** |
| --- | --- | --- |
| **Combined** | 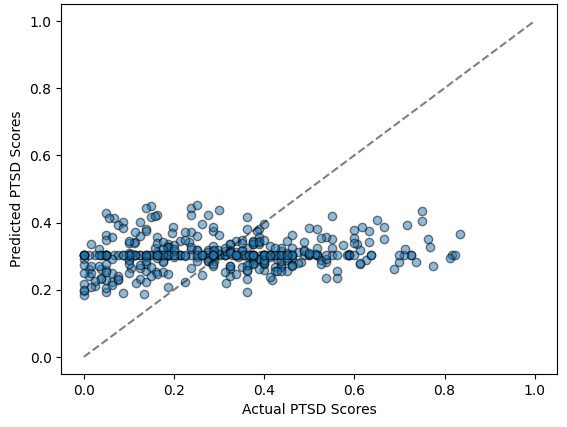 | 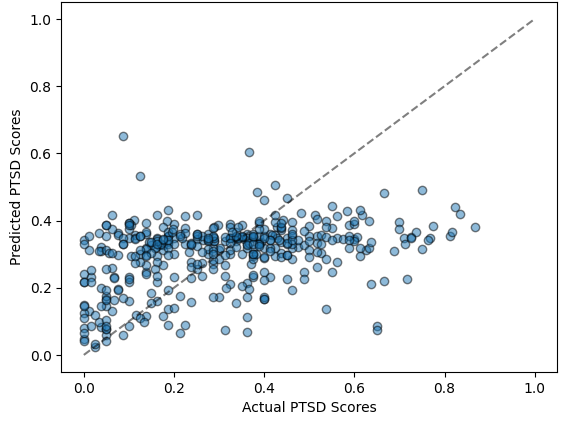 |
| **U.K.** | 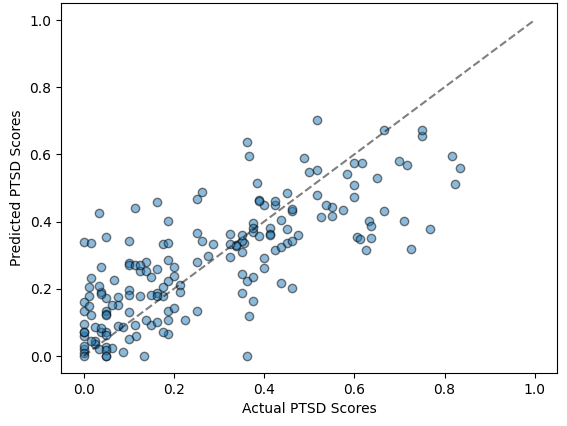 | 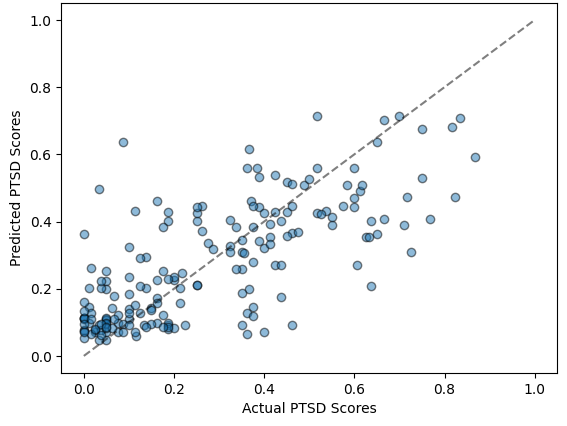 |
| **S.A.** | 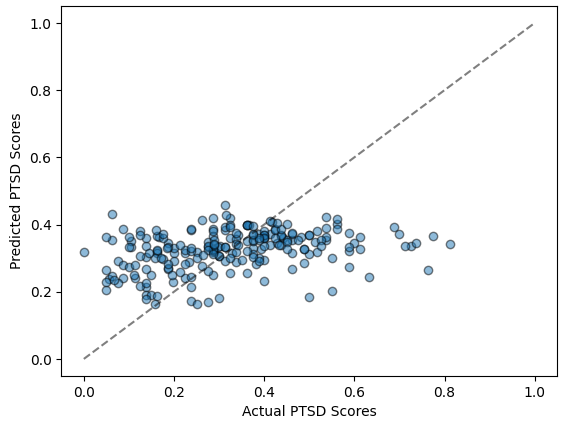 | 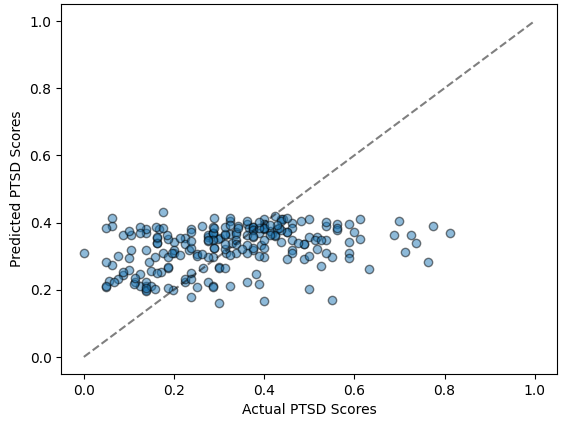 |

**Figure S5.** Feature importance plots for the LASSO (on the left) and XGBoost (on the right) models trained on the HIC dataset. The plots display the top 20 features contributing to PTSD prediction.


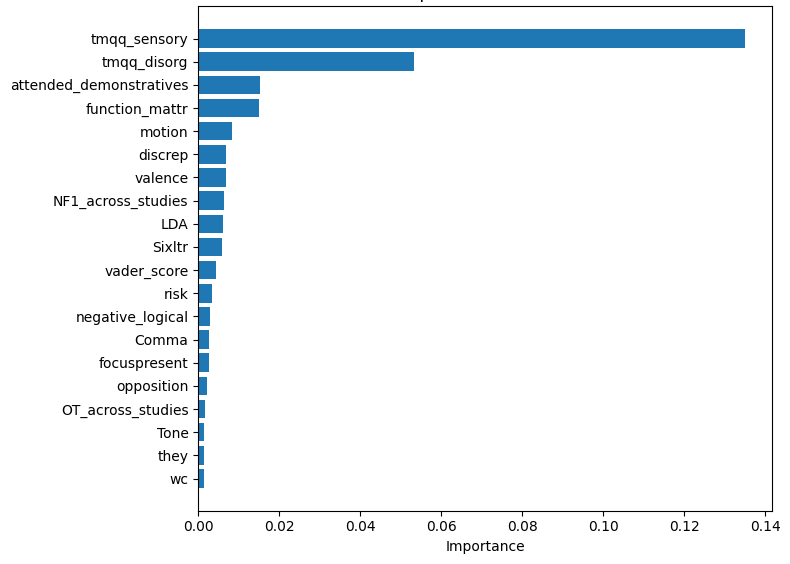

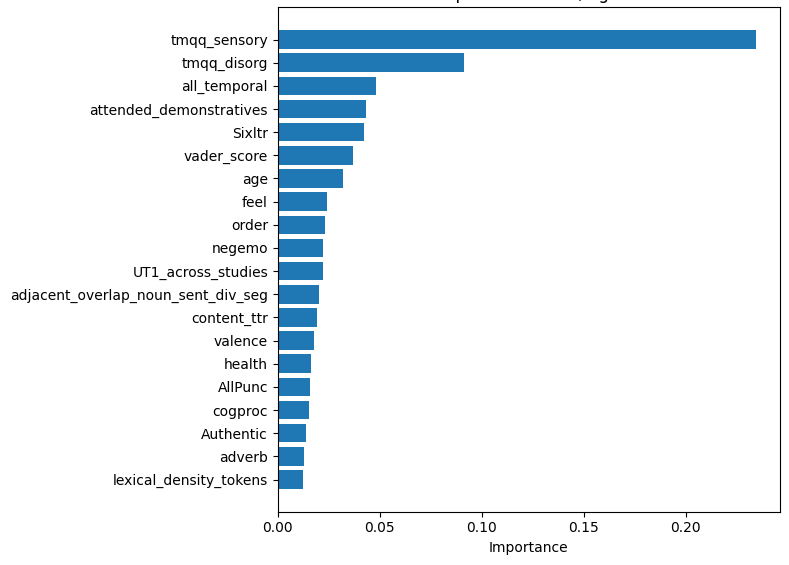


**Figure S6.** Feature importance plots for the LASSO (on the left) and XGBoost (on the right) models trained on the South African dataset. The plots display the top 20 features contributing to PTSD prediction.


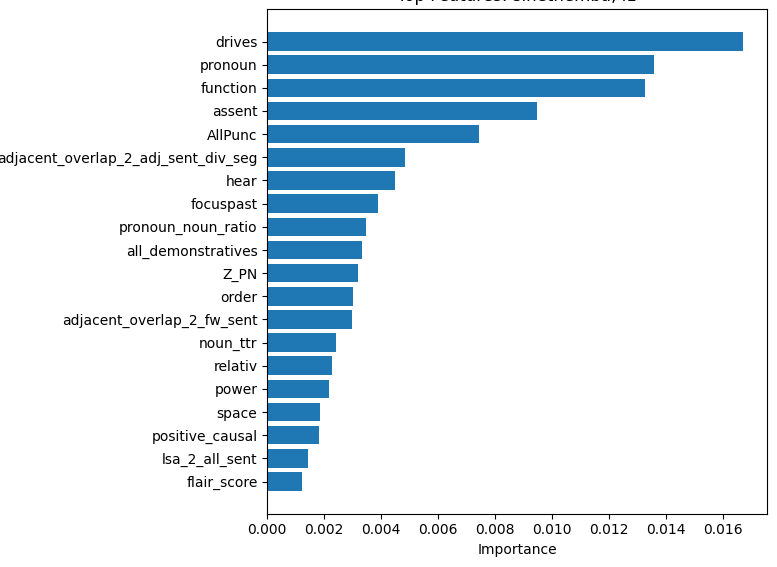

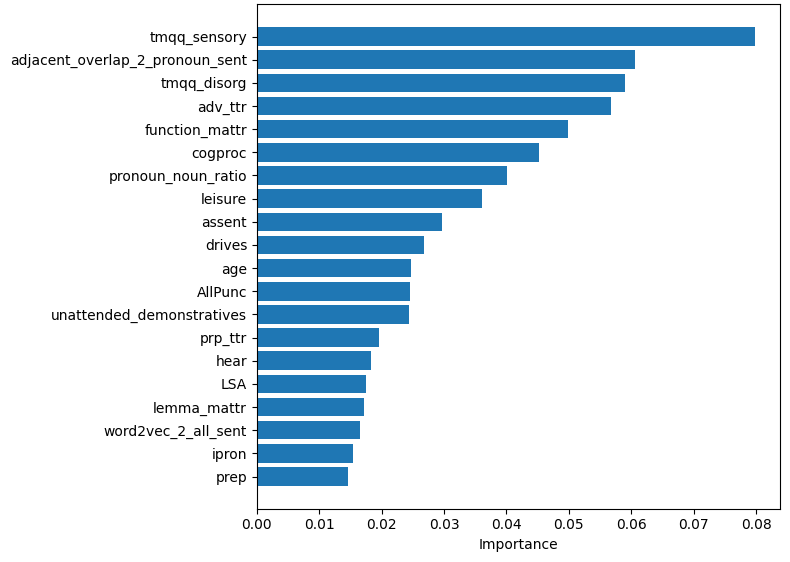


**Figure S7.** Feature importance plots for the LASSO (on the left) and XGBoost (on the right) models trained on the combined dataset. The plots display the top 20 features contributing to PTSD prediction. This outcome reflects the fact that the LASSO model trained on the combined dataset achieved zero correlation between predicted and observed PTSD scores, underscoring its inability to capture meaningful predictive patterns.


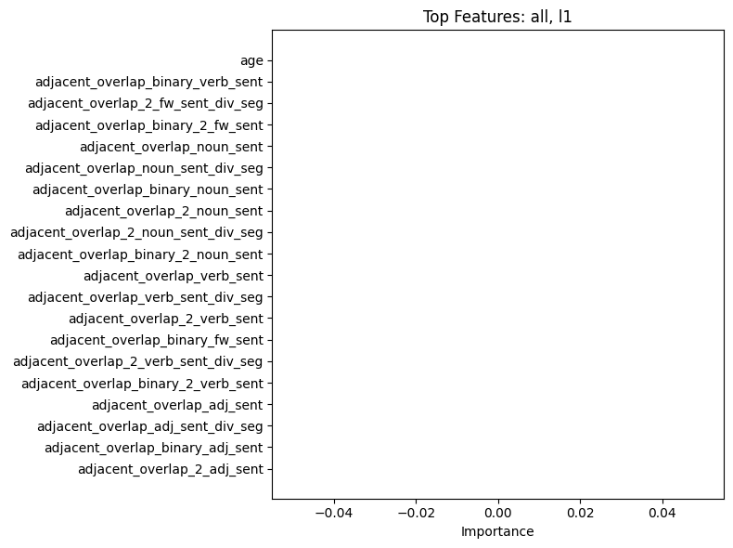

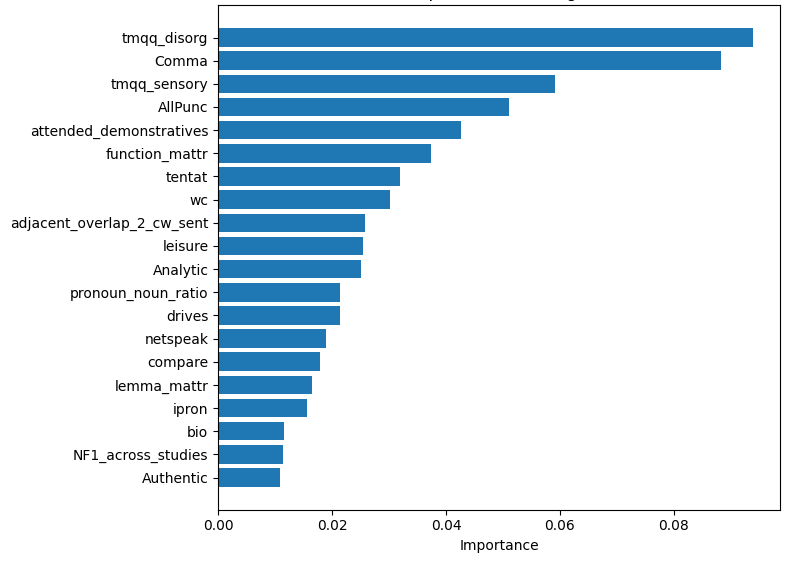

Supplement: Supplemental Material [file ZEPT_A_2589709_SM8083.docx]
